# Natural selection according to Darwin: cause or effect?

**DOI:** 10.1007/s40656-022-00485-z

**Published:** 2022-04-11

**Authors:** Ben Bradley

**Affiliations:** School of Psychology, Charles Sturt University, 164 George Street, Bathurst, NSW 2795 Australia

**Keywords:** Agency, Darwinism, Development, Historiography, Phenotype, *Vera causa*

## Abstract

In the 1940s, the ‘modern synthesis’ (MS) of Darwinism and genetics cast genetic mutation and recombination as the source of variability from which environmental events *naturally select* the fittest, such ‘natural selection’ constituting the cause of evolution. Recent biology increasingly challenges this view by casting genes as followers and awarding the leading role in the genesis of adaptations to the agency and plasticity of developing phenotypes—making natural selection a consequence of other causal processes. Both views of natural selection claim to capture the core of Darwin’s arguments in *On the Origin of Species*. Today, historians largely concur with the MS’s reading of *Origin* as a book aimed to prove natural selection the *cause* (*vera causa*) of adaptive change. This paper finds the evidence for that conclusion wanting. I undertake to examine the context and meaning of all Darwin’s known uses of the phrase *vera causa*, documenting in particular Darwin’s resistance to the pressure to prove natural selection a *vera causa* in letters written early in 1860*.* His resistance underlines the logical dependence of natural selection, an *unobservable* phenomenon, on the causal processes producing the *observable* events captured by the laws of inheritance, variation, and the struggle for existence, established in Chapters 1–3 of *Origin*.

Since the synthesis of Darwinism and Mendelian genetics in the 1930s and 1940s, Darwinians have not regarded the struggle for existence as a cause of natural selection. (Radick, 2009, p. 162).

They admit variation as a *vera causa* in one case, they arbitrarily reject it in another, without assigning any distinction in the two cases. The day will come when this will be given as a curious illustration of the blindness of preconceived opinion. (Darwin, 1859a, p. 423).

## Introduction

Before the seminal marriage of evolutionary theory with modern genetics, Gregor Mendel was commonly thought non-Darwinian—because the effects of genetic mutations were held to be discontinuous, and so incompatible with Darwin’s evolutionary dictum that ‘nature does not make jumps’ (e.g. Bateson, 1909; cf. Darwin, 1859a, pp. 171ff: ‘natura non facit saltum’). As soon as the ‘modern synthesis’ (MS; Huxley, 1942) gained influence over evolutionary science, its deployment of population genetics meant Mendel was recast as a Darwinian (Fisher, 1936; Sapp, 1990).

Like transformations have moulded understandings of Charles Darwin’s own work. Before the 1930s, his writings had played divers roles across different branches of science: biology; genetics; geology; biometrics; and taxonomy amongst them (e.g. Depew & Weber, 1994; Gayon, 1992). Come the MS, however, and Darwin got recast as the purveyor of a single idea—‘the best idea anyone has ever had’—meaning that, by century’s end, Darwinism had become synonymous with a belief in natural selection which was, purportedly, ‘the fundamental mechanism responsible’ for evolution (e.g. Dennett, 1995, pp. 21, 46). As a result, Darwin’s many other observations and theses about how evolution worked were side-lined as wrong or irrelevant to contemporary science. At the same time, the MS endowed Darwin’s treatment of natural selection with a newly-narrowed, and still-dominant identity (Provine, 1988; Smocovitis, 1992). It became the *cause* or *mechanism* of evolution: chance environmental events blindly winnowing random genetically-caused variations in organisms’ DNA, so that descendant gene pools (and, *only consequently*, organisms) grow better adapted to their conditions of life than were their ancestor populations.

Syncing nicely with the MS, by the year 2000 historians of science had firmly established an MS-consistent reading of *On the Origin of Species* (Darwin, 1859a; henceforth ‘*Origin*’) as a book primarily aimed to prove that ‘natural selection’ is the causal mechanism of evolution (e.g. Hodge, 2013; Hull, 2003; Pence, 2018; Ruse, 2005; Waters, 2003). Yet, over recent decades, biology’s MS has increasingly been challenged, qualified, or ‘extended’ by the findings of evolutionary science (e.g. Laland et al., 2015; Oyama et al., 2001; Walsh, 2015; West-Eberhard, 2003). In the process, scientists’ views have begun to diverge about the central thesis of *Origin* and, in particular, about how Darwin understood ‘natural selection.’ Such divergence presents a challenge to historians’ readings of *Origin* as purveying a view of evolution consonant with the MS. Does Darwin’s masterwork genuinely—but wrongly, according to a growing number of twenty-first century evolutionary biologists—equate natural selection to a ‘mindless, purposeless, mechanical process,’ as both modern synthesisers and their critics continue to claim (e.g. Dennett, 1995, p. 34; Lewontin, 1983, p. 275; Pigliucci et al., 2010, p. 11)? In which case evolutionary biology is en route to becoming *non*-Darwinian. Or do historians’ MS-consistent readings of *Origin* miscast its arguments?

This essay tackles those questions. It aims to reassess historical evidence about Darwin’s take on the argument that natural selection provides the causal mechanism for evolutionary change. I will examine in detail: how Darwin himself used the phrase ‘*vera causa*’; the way *Origin* constructs its argument about the causes of evolution; and how Darwin defended that book against criticisms of his approach to scientific investigation. I also review the grounds for modern historiographic conclusions that *Origin* argues natural selection to be a causal mechanism. In this, I draw out conflicting uses of that ambiguous phrase ‘*verae causae*’ by Darwin scholars. I conclude by showing that understanding natural selection as an effect of other processes—not a cause in its own right—has critical significance for contemporary evolutionary theory. To that end, I will start my argument by briefly outlining the place of understandings of Darwin’s work in debates about the explanatory status of natural selection in today’s evolutionary science.

## The contemporary scientific debate about natural selection

Twenty-first century science poses three kinds of challenge to MS understandings of evolution, under the banners: ‘evo-devo’; ‘developmental systems theory’ (DST); and a phenotype-first theory of adaptive change as led by ‘developmental plasticity.’ Despite their considerable differences (Bradley, 2020, pp. 97–101), these new approaches have together been dubbed the ‘extended evolutionary synthesis.’ But they do not all challenge the idea of natural selection as cause (e.g. Laland et al., 2013; Pigliucci et al., 2010). A subset of these approaches, which *do* consistently challenge the causal interpretation of natural selection, have been dubbed, ‘developmental,’ ‘situated’ and ‘active’ Darwinism’ (Noble, 2020; Walsh, 2012, 2015).

Evolutionary developmental biology (‘evo-devo’) addresses ‘the profound neglect of development in the standard modern synthesis framework of evolutionary theory’ (Muller, 2007, p. 943). It seeks to understand both how developmental processes have evolved and how they may have helped cause the evolution of adaptations (Arthur, 2002). But evo-devo typically retains what its advocates call ‘Darwin’s’ conception of natural selection: as a causal ‘process’ or ‘mechanism’ which ‘acts’ at the population level (e.g. Arthur, 2002, pp. 759–762; cf. Hall, 2012, p. 187; Muller, 2007, p. 94). DST typically theorizes phenotypic adaptation as developed via a system which incorporates an organism’s or population’s genes *along with* their ‘environments’—the term ‘environments’ including the ‘internal’ intra-cellular environment’s control of gene expression (epigenetics), plus developmental processes, alongside stable features of the ‘external’ environment (Oyama et al., 2001, pp. 1–11). However, DST still confusingly casts natural selection *both* as an ‘emergent phenomenon’ (a higher-order effect?) stemming from ‘lower-level’ processes, *and* as *a productive cause* of adaptation (Griffiths & Gray, 2001, p. 214; Weber & Depew, 2001, pp. 244–249). Finally, West-Eberhard (2003, pp. 33ff; Walsh, 2010) has advanced a comprehensive, evolutionary *theory of the phenotype* which conceptualises phenotypic development in terms of ‘plasticity’: the ability of an organism to react to an internal or external environmental input with reversible or irreversible changes ‘in form, state, movement, or rate of activity.’ West-Eberhard’s understanding of plasticity incorporates a stress on the agency of whole organisms, a theme that has become increasingly prominent over recent years (e.g. Bradley, 2020; Nicholson, 2014; Nicholson & Dupré, 2018; Noble, 2020; Walsh, 2015). She argues agentic plasticity ‘leads’ evolutionary change, with genes acting as ‘followers’ which subsequently stabilise adaptive phenotypic changes (West-Eberhard, 2003, pp. 157–158). Her analysis makes natural selection an effect of other causes.

These new approaches expose a rift between two competing views of evolution by natural selection: the eighty-year-old gene-stressing MS and its derivatives (e.g. Dennett, 1995; Huxley, 1942), versus those contemporary views which stress the plasticity of organismic agency, and of phenotypic development, as what guides adaptation. Yet *both* these rivals claim direct descent from *Origin*. So we find Dawkins (2006, p. xv, my italics) maintaining that his MS-based selfish-gene view of evolution ‘*is Darwin's theory*, expressed in a way that Darwin did not choose but whose aptness, I should like to think, he would instantly have recognized and delighted in.’ Meanwhile, the new ‘developmental’ paradigm of evolutionary biology, writes Walsh (2010, p. 336), ‘preserves more of the core of the *Origin of Species* than Modern Synthesis Replicator theory does’ (see too West-Eberhard, 2003, pp. 186–193; 2008). In particular, Walsh (2012, p. 192, my italics; drawing on West-Eberhard, 2003) argues that a crucial difference between these two Darwinisms is that, while the MS holds natural selection to be a causal force in its own right, developmental or active Darwinism cleaves closer to the *Origin* in making natural selection—*not* a cause of evolution, as per the MS view—but ‘a higher-order *effect*’ of a number of *other* causal processes, most notably, of the struggle for existence and of individual development.

Which poses a question: which of today’s two Darwinisms better captures what *Origin* argues?

## *Origin*’s framing of natural selection and Darwin’s responses to its critics

The explanatory status of natural selection in *Origin* does not just concern today’s historians. It worried Darwin himself, particularly in the few months following his book’s launch. Several reviewers—including Darwin’s allies Charles Lyell (1859), Thomas Huxley (1860a, 1860b) and Asa Gray (1860)—had quickly queried the scientific orthodoxy of the book’s method of argument, and the comprehensiveness of its evidence for its conclusions. Darwin took pains to address these worries, not just in private letters, but in amendments to later editions of *Origin*, and published defences of its arguments.

In the first edition of *Origin*, Darwin (1859a, pp. 84, 146) habitually wrote as if natural selection were an intelligent agent, ‘intently watching each slight alteration’ in an organism’s structure and habits, so that it could ‘pick out with unerring skill each improvement’:

It may be said that natural selection is daily and hourly scrutinising, throughout the world, every variation, even the slightest; rejecting that which is bad, preserving and adding up all that is good; silently and insensibly working, whenever and wherever opportunity offers, at the improvement of each organic being in relation to its organic and inorganic conditions of life.

Writing in this vein, Darwin (1859a, pp. 85, 156) cast natural selection as a ‘power,’ which ‘acts by life and death,’ and so ‘causes’ extinction, for example. Not only antagonists (like Adam Sedgwick, 1859), but even allies like Charles Lyell (1860a) and Joseph Hooker (1860) complained Darwin had cast natural selection as a power akin to a deity, a ‘deus ex machina’ as Hooker (1862) later put it. Darwin denied the claim. (And later editions of *Origin* qualified his use of anthropomorphic language.)^1^ Yet both the rhetorical organisation of his argument, and the fact that his book used an ordinary language immanently ‘imbued with intentionality,’ weakened these denials (Beer, 2000, p. 81).

Darwin had launched *Origin*’s argument with an account of ‘variation under domestication’ which celebrated the considerable changes in the forms of domesticated plants and animals (especially pigeons) effected by breeders’ powers of ‘artificial selection.’ He then extended this analogy to provide the framework for his exposition of ‘natural selection.’ In so doing, he rhetorically projected an image of ‘nature’s power of selection’ in the form of a human skill, though one of ‘far higher workmanship’ and producing modifications ‘infinitely better adapted to the most complex conditions of life’ than the ‘feeble’ ‘artificial’ efforts of stud-farmers and horticulturalists (Darwin, 1859a, pp. 84, 109). At the same time, when pushed, he strongly resisted the idea that ‘natural selection’ *was* an anthropomorphic causal agency (Young, 1971).

This contradiction arose because, as Beer (2000, pp. xviii, 3, 48) put it, *Origin*’s central argument ran directly ‘against the grain of the language available’ to Darwin as a Victorian man of science, a language which spot-lit ‘design and creation.’ Darwin’s struggle was to portray natural law and the uniformity of nature as things *opposed to* design and divine creation. Thus, viewed in the round—and despite the vagaries of the metaphorical way he had expressed his thesis—his book aimed to convince readers that natural selection was ‘*one general law*, leading to the advancement of all organic beings, namely, multiply, vary, let the strongest live and the weakest die’ (Darwin, 1859a, p. 244, my italics; cf. Darwin, 1874, pp. 48, 613). In 1861, he hastened to underline this aim by adding a caveat to the third edition of *Origin* (1861a, p. 85, my italics):

Several writers have misapprehended or objected to the term Natural Selection. Some have even imagined that natural selection induces variability, whereas it implies only the preservation of such variations as occur and are beneficial to the being under its conditions of life … It has been said that I speak of natural selection as an active power or Deity; but who objects to an author speaking of the attraction of gravity as ruling the movements of the planets? Every one knows what is meant and is implied by such metaphorical expressions; and they are almost necessary for brevity. So again it is difficult to avoid personifying the word Nature; but I mean by Nature, only the aggregate action and product of many natural laws, and by laws the *sequence of events as ascertained by us*. With a little familiarity such superficial objections will be forgotten.

Natural selection, Darwin here re-asserts, describes a ‘sequence of events as ascertained by us.’ It does not *actively produce* variations: it *results from* (the preservation of) variations—such preservation being something which itself ‘*results from* the struggle for existence’ (Darwin, 1859a, pp. 5, 433, my italics).

This reading of *Origin* is reinforced by Darwin’s response to a fierce debate over the explanatory status of ‘natural selection’ in the early months of his book’s life. *Origin* itself labels natural selection in several ways, most commonly as a principle, a law, or a theory. Within weeks of its appearance, however, both private correspondents and public reviews opened a debate about the causal efficacy of natural selection. Had Darwin’s book proved natural selection to be a *true cause* (or ‘*vera causa*’) of the origination of species? His friends Huxley (1860a, 1860b) and Gray (1860), while both hugely appreciative of *Origin*, concluded that Darwin had failed to prove natural selection was the effective cause of evolution. Others were more contemptuous. Palaeontologist Richard Owen (1860) pronounced natural selection a hypothesis resting on ‘a purely conjectural basis.’ Philosopher of science John Herschel dismissed *Origin* as ‘the law of higgledy-piggledy’ (Darwin, 1859b; Hull, 2003, pp. 181–182). And Darwin’s old geological mentor Adam Sedgwick (1860, p. 335) found in *Origin* a ‘baseless theory.’

Darwin (1863a) reacted to these criticisms by pointing out in a letter to the *Athenaeum* (provoked by Owen, 1863) that, though he continued to believe that ‘the theory, or hypothesis, or guess, if the reviewer likes so to call it, of natural selection’ provided the best explanation for the origin of species, such explanation ‘signifies extremely little in comparison with the admission that species have descended from other species and have not been created immutable.’ In short, the chief aim of his book was to win scientific acceptance of the fact that all species of creature were bound together by a web of affinities that comprised a ‘community of descent.’ His exposition of natural selection was first and foremost, aimed to gain assent for the *fact* of evolution. Its exact explanatory status was of secondary importance.

Meanwhile, behind the scenes, Darwin worked hard to garner evidence which would satisfy Huxley’s doubts about the explanatory status of natural selection. Huxley (1860b, pp. 74–75) did not himself use the term ‘*vera causa*’ in his review of *Origin*. His argument was that Darwin’s thesis on natural selection would remain ‘a hypothesis,’ and not yet a ‘theory of species,’ until ‘positive evidence’ could be produced that a group of animals (or plants) had, ‘by variation and selective breeding, given rise to another group which was, even in the least degree, infertile with the first.’ Besides conducting many (unsuccessful) experiments of his own to prove the sterility of inter-breed hybrids in flowers, Darwin recruited a host of zoologists, botanists and horticulturalists to find evidence to fill the gap that Huxley had identified, including Hooker, Muller, Tegetmeier, Gray and many others—even sending an open letter to the readers of the *Journal of Horticulture* to beg for relevant facts (Darwin, 1862). The chapter on hybridism in the fourth edition of *Origin* (Darwin, 1866) was expanded to discuss the most promising new findings, with the hope of satisfying Huxley. The chapter on hybridism in *The Variation of Animals and Plants under Domestication* (Darwin, 1868) also aimed to answer Huxley’s criticism. And later, the opening paragraphs of the last chapter in the final edition of *Origin* (Darwin, 1876) were enlarged to address the sterility of hybrids. Huxley remained unconvinced.

But Darwin’s strongest and most immediate response to critics of the scientific status of *Origin*’s claims was directly to contest the need to prove natural selection a *vera causa*. Within three months of the book’s publication, he told his closest friend Hooker (Darwin, 1860a) that Huxley ‘rates higher than I do the necessity of Natural Selection being shown to be a vera causa always in action.’

### Darwin contests the need to prove natural selection a *vera causa*

Three kinds of consideration underlined the subordinate importance of the *vera causa* criterion for Darwin. Firstly, the exact nature of the standard of proof for a *vera causa* had been disputed so much over previous decades that, by 1859, its meaning was extremely loose (Ruse, 1975). *Origin* (Darwin, 1859a, p. 423) lampoons the resultant imprecision in scientific identifications of natural phenomena as *verae causae*: while ‘several eminent naturalists … admit variation as a *vera causa* in one case, they arbitrarily reject it in another, without assigning any distinction in the two cases’—such arbitrary identifications revealing only the power of ‘the blindness of preconceived opinion.’

The inconsistency of scientific judgements about *verae causae* that Darwin disparaged is confirmed by three trail-blazing historiographical essays (published in the 1970s) which concluded that *Origin* aimed to prove natural selection a *vera causa*, in that all three disagree about what Darwin would need to have done to achieve this aim (Hodge, 1977, 1989; Hull, 1973, 2003; Ruse, 1975, 1999, p. 57). This disagreement continues today, as Sect. 4 of this paper documents. One historian, Greg Radick (2002, p. 13, my italics), has even glossed Darwin’s adherence to the *vera causa* ideal as meaning that *Origin* sought to show that ‘*the causes that together produced natural selection*—variation, inheritance and the struggle for existence—were all “true causes,” that is, causes evidenced independently of the facts they were held to explain.’ Radick’s reading—that *Origin* frames natural selection as *the consequence* of several other, directly-observable (and hence ‘true’) causal processes—points directly to the argument I advance here. But it stands in stark conflict with the views of the *Origin*’s strategy elaborated by Hodge and Ruse in the 1970s, as well as many more recent historiographic claims.

Secondly, Darwin’s own most-repeated criterion for the scientific reality of natural selection was that it could explain the several distinct ‘large classes of facts’ that *Origin* (1876, p. 568) argued natural selection did explain. This standard of proof was akin to what William Whewell (1840) called a ‘consilience of inductions’: ‘the best kind of science … comes when different areas of science are brought together and shown to spring from the same principles’ (Ruse, 1975, p. 163). Yet the status of such consilience vis-à-vis *verae causae* remains uncertain. According to some historians, such ‘consilience’ was a hallmark of *verae causae* (Ruse, 1975, 1999, p. 58; Waters, 2003). Others, like Hodge (1989, pp. 171–173, my italics) argue that Whewell ‘offered his consilience ideal as *an alternative*’ to the *vera causa* ideal.

The ‘large classes of fact’ or ‘different areas of science’ which *Origin* (1859a, pp. 415, 420; 1876, pp. 137, 424) treats as explained by ‘the same principle’ of natural selection include: the homologous forms of rudimentary, embryological, and anatomical structures in taxonomically related species (e.g. wing of the bat, fin of the porpoise, leg of the horse, human hand); the fact that pre-evolutionary taxonomic classification could be arranged within ‘a few great classes, in groups subordinate to groups, and with the extinct groups often falling in between the recent groups’; endemic species on oceanic islands being related to the nearest source of immigrant species (as in the Galapagos archipelago); the gradual diffusion of dominant forms in the geological record; the co-adaptations of different species to each other within the same habitat; and the lack of perfection of some adaptations—‘the sting of the bee, when used against an enemy, causing the bee’s own death,’ ‘drones being produced in such great numbers for one single act, and being then slaughtered by their sterile sisters,’ ‘the astonishing waste of pollen by our fir-trees’ (Darwin, 1876, pp. 415, 419).

Darwin reverted to his consilient criterion of proof time and again, not just in *Origin*, but in his other books (e.g. Darwin, 1874, p. 24; 1890, p. 113), and in his letters. For example: ‘It seems to me that an hypothesis is developed into a theory solely by explaining an ample lot of facts’ (Darwin, 1860b). In contrast, Darwin’s publications *never* refer to natural selection as a *vera causa* (nor do they ever refer to it as a mechanism; Ruse, 2005). In fact *Origin* was the only one of his books to reference *verae causae* at all. It uses the phrase thrice: once to refer to ‘community of descent’; once to suggest that, when a single species occurred at ‘several distant and isolated points,’ the ‘the *vera causa* of ordinary generation with subsequent migration’ was a better explanation for it doing so than the ‘miracle’ of several separate divine creations; and once, as we just saw, to ridicule the arbitrariness of the assignment to ‘variation’ of the status of a *vera causa* in the creationist arguments of ‘several eminent naturalists’ (Darwin, 1859a, pp. 159, 352, 482). These three usages all occur in polemics directed at creationists—the last stating directly that naturalists’ identification of *verae causae* owed more to prejudice than to science. Which might suggest the phrase functioned at best rhetorically in *Origin*, which variously enlisted, and questioned, its gravitas as a shibboleth of scientific proof.

Thirdly, three months after *Origin* came out, Darwin discovered a powerful parallel between natural selection and Newton’s law of gravity. In February 1860, Darwin was reading David Brewster’s (1855) *Memoirs of Sir Isaac Newton*. This was the month he was most acutely focused on arguments, like Huxley’s (1860a), that *Origin* had failed to prove natural selection causally efficacious. Brewster (1855, pp. 282ff) recounted how, in 1710, Leibnitz had attacked Newton’s theory of gravity as ‘introducing occult qualities and miracles into philosophy.’ Newton retorted that the theory of gravity was:

proved by mathematical demonstration, grounded upon experiments and the phenomena of nature; and that to understand the motions of the planets under the influence of gravity, *without knowing the cause of gravity*, is as good a progress in philosophy as to understand the [movements of the clockwork of a clock, as a clockmaker does] without knowing the cause of the gravity of the weight which moves the machine … (Newton, 1711, quoted in Brewster, 1855, p. 283, my italics)

Darwin pounced on this passage because it underlined a distinction between *law* and *cause,* as Darwin swiftly pointed out to Lyell and Gray. Darwin’s (1860c) comment to Lyell was that, though Leibnitz held the law of gravity to be unscientific, mysterious or ‘occult’ (because gravity had not been directly observed), Newton’s law nonetheless added to our knowledge because it explained ‘the movement of wheels of clock, *though the cause of descent of the weight could not be explained*,’ adding to Gray the next day (Darwin, 1860d): ‘This seems to me rather to bear on what you say of Nat. selection not being proved as a vera causa.’

What interested Darwin about Newton’s reply to Leibnitz was that the law of gravity brings under one descriptive formula various ‘sequences of event as ascertained by us’—tidal flows, falling apples, clockwork, and planetary orbits—even though Newton could not say *what caused* those events.^2^ Likewise, the origin of new species by natural selection was too slow to be observed. Yet, like Newton’s law, Darwin’s argument gave coherence to various ‘sequences of event’—palaeontological succession of types, geographical distribution, taxonomic nesting, homologies of anatomical structures in related taxonomic classes, in rudimentary organs and in embryos etc.—however much *what caused* those events remained open to question.

In the same month that he was reading Brewster, Darwin (1860e) developed a second parallel with physics, this time between his ‘hypothesis’ of natural selection and the wave or ‘undulatory’ theory of light, which, by the 1850s, was becoming increasingly favoured over Newton’s (1704) corpuscular theory. Of course, no one had ever observed the undulations in the so-called ‘luminiferous ether’ which constituted light, according to Robert Hooke (1664) and Christiaan Huygens (1690). Yet, said Darwin, the wave theory ‘groups together & explains a multitude of phenomena,’ such as the interference patterns seen in Thomas Young’s (1804) diffraction experiment, and so was ‘universally now admitted as the true theory.’ ‘The undulatory theory of Light *is very far from a vera causa*,’ noted Darwin (1860f, my italics), yet it was scientifically ‘allowable (& a great step) to invent the undulatory theory of Light.’ So why should not scientific procedure allow Darwin (1860g) also to ‘invent [the] hypothesis of natural selection … & try whether this hypothesis … does not explain (as I think it does) a large number of facts in geographical distribution—geological succession—classification—morphology, embryology &c. &c.’?^3^ By implication, Darwin is here acknowledging that natural selection, while having scientific value, is also ‘very far from a vera causa.’

Darwin’s parallel between the law of gravity and natural selection was swiftly spliced into the last chapter of the third edition of *Origin* (1861a, pp. 514–515), which thenceforth noted that, though ‘the law of the attraction of gravity’ had been attacked by Leibnitz because no one knew ‘what is the essence of the attraction of gravity,’ yet ‘no one now objects to following out the results consequent on this unknown element of attraction.’ *Origin*’s final edition saw a further inclusion—Darwin’s (1876, p. 421) parallel between natural selection and the wave theory of light—a parallel which had already been developed at greater length in the exposition of natural selection opening *Variation* (Darwin, 1868, vol.1, pp. 8–9).^4^

## Modern *vera causa* readings of *Origin*

I now consider how my reading of *Origin* bears on evidence for contemporary historians’ conclusion that the main aim of *Origin* is to prove natural selection has been the ‘true cause’ (*vera causa*), ‘mechanism’ or ‘causal force’ effecting the origin of species (e.g. Gildenhuys, 2004; Hodge, 2013; Pence, 2018; Ruse, 2005). It should be remembered that, whilst *vera causa* interpretations of *Origin* are widely assumed by today’s Darwin scholars, they disagree amongst themselves as to what *vera causa* might have meant to Darwin in 1859. Pence (2018) finds seven current historiographic interpretations of the philosophy of science underpinning *Origin*—and his list is not exhaustive.

Neither Darwin nor *Origin* ever claim that natural selection is a *vera causa*—though, as we saw above, *Origin* does use this phrase in connection with three other facets of Darwin’s argument. Nor does Darwin anywhere, in his publications, notebooks or private correspondence, say that the book was *designed* to prove natural selection is a *vera causa*. On the contrary, he said the book was designed to prove that all species share in a community of descent (besides which, he added, the validity of any claims he had made about natural selection signified ‘extremely little’; Darwin, 1863a, see Sect. 3).

So: what evidence do today’s historians advance to back their contention that Darwin wrote *Origin* to prove natural selection a *vera causa*? They largely ignore the evidence I reviewed in Sect. 3.1, quoting instead the first third of the postscript to a letter Darwin wrote to botanist George Bentham, in May 1863. This letter concerned an address, intended to support *Origin*, which Bentham was preparing to give to the Linnaean Society (of which he was president) in two days’ time. Bentham (1863, my italics) was worried that Darwin’s theory could not explain why some species of ‘northern hemisphere’ plants were found in Tasmania and Australia’s Victorian Alps to ‘have gone through so many thousand generations in both hemispheres *unaltered*,’ whilst other species of such plants had changed so much as to become almost unrecognisable. The postscript to Darwin’s (1863b) reply reads as a kind of executive summary to help the doubting Bentham prepare his imminent address by clarifying three alternative kinds of grounds upon which a belief in natural selection could be based:

In fact the belief in natural selection must at present be grounded entirely on general considerations. (1) on its being a vera causa, from the struggle for existence; & the certain geological fact that species do somehow change (2) from the analogy of change under domestication by man’s selection. (3) & chiefly from this view connecting under an intelligible point of view a host of facts.— When we descend to details, we can prove that no one species has changed: nor can we prove that the supposed changes are beneficial which is the groundwork of the theory. Nor can we explain why some species have changed & others have not …^5^

A century later, in 1975, Ruse concluded that this postscript shows that, useful though Darwin believed the analogy between artificial and natural selection might be (Darwin’s 2nd point), ‘the chief proof for Darwin of the truth of his theory was that it had explanatory power in all of these many diverse areas’ (Darwin’s 3rd point). In a footnote to the same article, Ruse (1975, p. 177) challenged the import of the postscript’s first point, saying that, although Darwin here ‘wrote of natural selection as a *vera causa*,’ by 1859 this term ‘was used almost as loosely as “deduction”.’ Elsewhere, however, both in his 1975 article and in later works, Ruse (1975, p. 175; 1999; 2005) agrees with Hodge (1977, p. 238) that in *Origin*, Darwin was ‘committed’ or ‘desperately keen’ to show that ‘his evolutionary reasonings were based on a *vera causa*, natural selection.’ Yet these two historians disagree as to whether Darwin’s ‘commitment’ was informed by John Herschel’s interpretation of Thomas Reid’s understanding of *vera causa* (Hodge, 1977, 1989), or by the incompatible views (according to Hodge, 1989, p. 172) of William Whewell (Ruse, 1975, 1999).

Hodge (1977, pp. 240–241; Hodge, 1989, p. 190; 2013, p. 2273) also disagrees with Ruse about Darwin’s postscript to Bentham. He reads its three points as a rationale for decoding *Origin*’s entire structure as something rooted in a three-step strategy to prove natural selection is a *vera causa* (a strategy which I will describe shortly). Gildenhuys (2004, pp. 594, 605) and Pence (2018) also ground their arguments on the postscript (though both disagree with Hodge’s reading). None of these articles by Ruse, Hodge, Gildenhuys, or Pence, which cite the postscript to Bentham, discusses the far more substantial correspondence Darwin had had in the opening months of 1860, regarding the causal status of natural selection (discussed in Sect. 3.1). Nor do they reference the changes Darwin made to the third and later editions of *Origin*, reflecting the lasting importance of the points made in that correspondence.

Whether in his publications, his notes or his correspondence, Darwin rarely mentioned *verae causae.* His only published mention of a *vera causa* prior to its appearance in *Origin* was twenty years earlier, in his geological ‘Observations on the Parallel Roads of Glen Roy’ (Darwin, 1839; see Sect. 5.1 below). This paper had over-confidently (and falsely) identified the action of river deltas flowing into the sea as a *vera causa* for the formation of the ‘buttresses’ found below Glen Roy’s ‘parallel roads,’ a deduction Darwin later accounted ‘a great failure, and I am ashamed of it’ (Darwin, 1958, p. 84). Bar *Origin*, none of his other books or papers mention *verae causae.* The sixty plus years of his vast correspondence contain just nine mentions of *verae causae.* Seven of these occur in the *vera causa* debate about natural selection during the five months after the book came out—all of which dispute ‘the necessity of Natural Selection being shown to be a vera causa always in action’ (Darwin, 1860a; see Sect. 3.1).

Darwin’s last ever use of the term *vera causa* is in the oft-cited postscript to Bentham. This comes more than three years after his earlier flurry of correspondence about the causal status of natural selection. By May 1863, Darwin (1861a, 1861b) had re-edited the text of *Origin* to underline his distinction between cause and law, and now believed that, among his scientific allies, there were many who had accepted natural selection was a *vera causa* (whatever that meant), including John Stuart Mill.^6^ He also knew that many other eminent men of science (including Huxley, Sedgwick, Owen, and Whewell) continued to deny it this status. So, when he noted to Bentham (a worried ally) that the belief in natural selection ‘must at present be grounded entirely on general considerations,’ he *did not*, in so many words, claim that natural selection *was* a *vera causa*. Because, as his whole letter underlined—and the much-quoted postscript reiterates^7^—he knew that there could be *no direct observational evidence* (‘details’) that even ‘one species has changed.’ Even for those who believed natural selection to be a *vera causa*, its status as such could only be *deduced* from things that *could* be observed (which, according to some interpretations of the concept, disqualified it as a *vera causa*, because, in the words of one critic, his theory was ‘not *inductive*—not based on a series of acknowledged facts’; Sedgwick, 1860, p. 334).

The postscript’s first point therefore acknowledges that, at best, natural selection’s status as a *vera causa* could only be derived *indirectly* from observational evidence for ‘the struggle for existence; & the certain geological fact that species do somehow change.’ It was for this reason that—though the analogy between artificial and natural selection provided another possible rationale—the postscript went on to stress that, *for Darwin*, the *chief* basis for a belief in natural selection was *not* that it was a *vera causa*, but *consilience*: its ‘connecting under an intelligible point of view a host of facts’ (see Sect. 3.1).

Claims that *Origin* is structured to prove natural selection a *vera causa* are further weakened by Hodge’s (e.g. 1977, p. 239; 1992; 2013) own efforts to force the book into the three-step framework he deems such a proof should take: ‘in explaining any phenomenon, one should invoke only causes whose *existence* and *competence* to produce such an effect can be known independently of their putative *responsibility* for that phenomenon.’ This implies, says Hodge, that *Origin* should comprise three sections, each section containing a group of chapters evidencing in turn: the *existence* of natural selection; the *competence* of natural selection to produce species-change; and, finally, evidence that natural selection really had been *responsible* for species-change. ‘Unfortunately,’ says Hodge (1977, pp. 238, 242), *Origin* ‘violates’ this structure, ‘misleadingly’ making ‘successive departures’ from it—departures which ‘were eventually enough to render the strategy and organization of his most famous book unhelpfully and quite unnecessarily obscure.’Which means the three ‘general evidential considerations’ upon which Darwin *should* have focused, ‘*do not* map onto the *Origin*’s three clusters of chapters’ (Hodge, 1977, p. 244; 2013, p. 2274, my italics).

## *Origin* on what effects natural selection

Modern debates about Darwin’s putative ‘commitment’ to the *vera causa* principle and his associated ‘epistemological self-consciousness’ (e.g. Hodge, 1977, p. 238; 2000, p. 29) attain a high degree of philosophical sophistication—far higher than any discussion to be found in Darwin’s own writings. Historiographers and philosophers of science make superfine distinctions between the epistemologies that are deduced to have influenced, or not to have influenced, Darwin’s authorial consciousness. Against this, Hull’s (1973, 2003) essays repeatedly demonstrate how shallow an understanding of epistemological issues—and the arguments of contemporary philosophers of science like Mill—Darwin (and Huxley) actually possessed.

As Darwin’s autobiography (1958, p. 140) candidly admitted, ‘my power to follow a long and purely abstract train of thought is very limited.’ And while he did pay some attention to metaphysical subjects in his twenties, his attitude to the topic had become increasingly jaundiced as his reading progressed. During 1838, he spent several months absorbing the opinions of Hume, Mackintosh, Abercrombie, Comte and Ferrier, all of whom belittled metaphysics as, for example, ‘a name of reproach and derision’ (Mackintosh, 1830, p. 4). By October of that year, he had concluded that: ‘To study Metaphysics, as they have always been studied appears to me to be like puzzling at astronomy without mechanics … we must bring some *stable* foundation to argue from’ (Darwin, 1838, p. 5). In later years his use of the term ‘metaphysical’ became derogatory.^8^ Books or articles that his letters dubbed ‘metaphysical,’ were condemned as ‘mere verbiage,’ being ‘barely intelligible,’ dealing in ‘far-fetched analogies,’ and constituting ‘rubbish’ produced by a ‘wind-bag’ with ‘muddled... brains’ and ‘an entire want of common sense’ (Darwin, 1845, 1857, 1860h, 1861b, 1864, 1871, 1874, p. 78).

Given the improbability that Darwin adhered to a refined philosophical understanding of what *vera causa* meant when he used the term, I now try to decode what it did mean to him. First, by emphasising the most obvious common denominator in the various conflicting philosophical understandings of *verae causae* current in his day. And second, by examining what Darwin assumed on the few occasions when he himself did mention *verae causae*.

Despite a slim evidential basis, modern Darwin scholars have imaginatively constructed several contrasting pedigrees for Darwin’s understanding of *verae causae*—whether via Thomas Reid (Hodge, 1989, p. 171), William Whewell (Ruse, 1975), John Herschel (Gildenhuys, 2004; Pence, 2018), or Charles Lyell (Rudwick, 2005; Sponsel, 2018). Whatever the merits of these different genealogies, all four hypothesised sources share one stress: the need for first-hand observational evidence in establishing the existence of a true cause. Reid insisted on ‘direct experiential acquaintance … as the only acceptable form of evidence for the known truth, reality or existence of a cause’ (Hodge, 1989, p. 169). Whewell (like Sedgwick and Huxley) objected to *Origin* because no-one could ‘adduce a single example of one species evolving in nature into another. Nor had plant and animal breeders, through all their efforts, succeeded in producing a single new species’ (Hull, 2003, p. 184). Herschel (1830, #138) held that the best way of establishing a *vera causa* was from ‘experience [showing] us the manner in which one phenomenon depends on another in a great variety of cases.’ And Lyell’s uniformitarianism leant on the argument that causes *which we can directly observe in the present*, like the slow action of coastal waves, can be used to reconstruct the vast prehuman past as recorded in rock strata, *so long as* we assumed that ‘the same causes … had been at work with the same intensities and in the same overall circumstances’ from the time the first rock formed through to the most recent (Hodge, 2000, pp. 28–29).

### Darwin’s own usage of ‘*vera causa*’

**Fig. 1:**
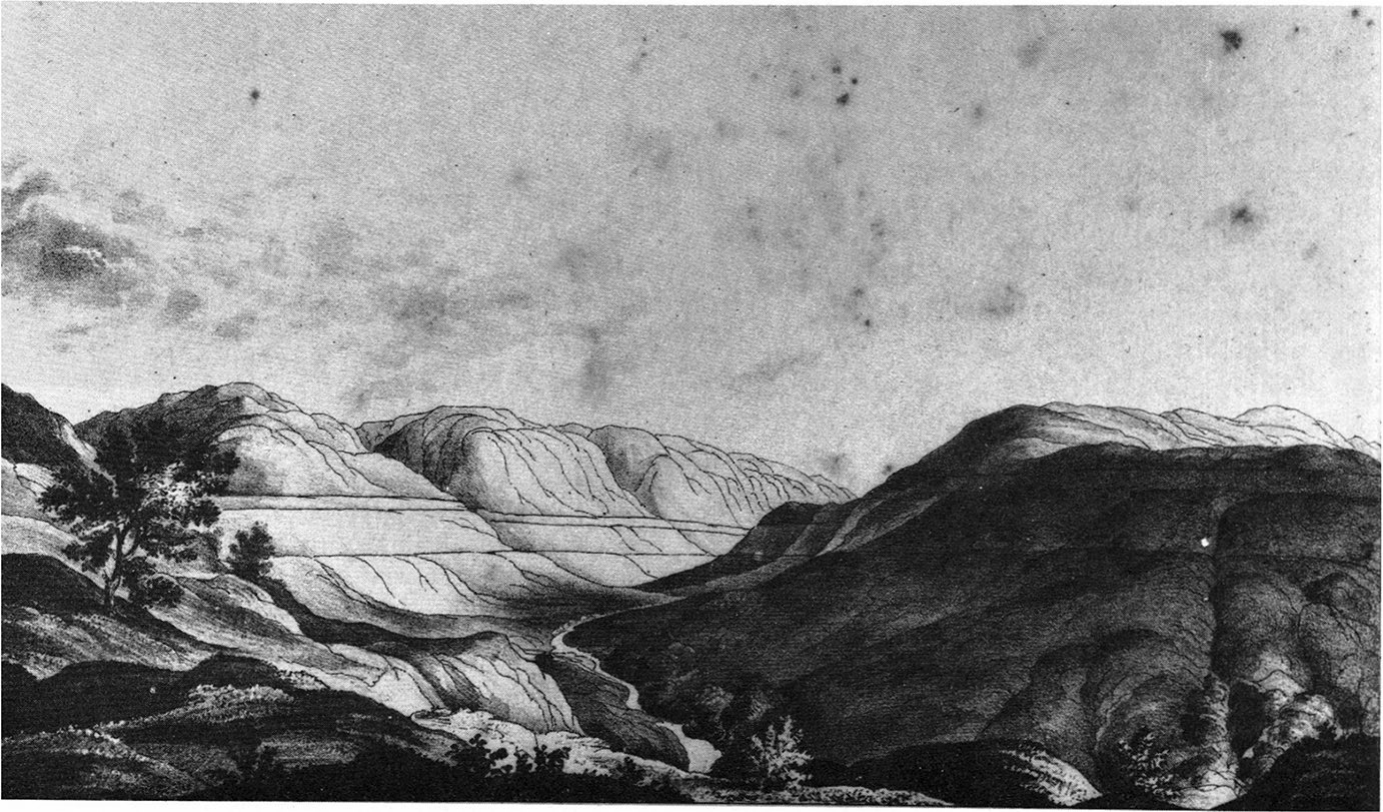
Darwin’s (1839) illustration of the parallel roads of Glen Roy. The ‘buttresses’ are depicted by bent lines which represent bulging piles of rocky debris (e.g. below the lowest of the three roads)

Turn to Darwin’s own usage of ‘*vera causa*’ and we also find a stress on direct observation. His ill-fated Glen Roy paper explains the ‘buttresses’—flat-topped accumulations of alluvial gravel and other debris *below* the lowest of the parallel roads (see Fig. 1) —as left-over ‘deltas’ made by rivers or ‘streamlets.’ These streamlets he deemed to have formed the deltas or ‘raised beaches’ of the roads themselves, at the level which the streamlets flowed into the sea *before* the Glen was tectonically raised above present-day sea-levels by a sequence of crustal uplifts. (Darwin argued that the problematic lower-level buttresses must be remains of alluvial deposits made by the deltas of these same streamlets after further, less dramatic, crustal liftings of the land.) In this way, he exploited the easy-to-observe fact of the alluvial action of river deltas, which was already a central plank of previous geological explanations for the parallel roads (Rudwick, 1974, pp. 106–107). Darwin’s paper (1839, p. 52) sums up this step in its argument by confidently asserting: ‘no one can doubt [t]hat this intervening cause [delta formation by rivers] has been … a *vera causa*.’

His only other non-*Origin*-related use of the phrase was in a letter to William Redfield in February 1840. Redfield (1839) had just published an article relating ‘a few cases in which whirlwinds of great activity and violence appeared to have resulted from the action of fires.’ Darwin, who had long puzzled over the origin of waterspouts seen on his *Beagle* voyage, added an observation to Redfield’s list. This regarded a whirlwind and waterspouts resulting from an island-forming, submarine, volcanic eruption, observed by a Captain Tilliard off the Azores in June 1811.^9^ Darwin (1840) wrote: ‘Taking your account of the whirlwinds produced by artificial fires, we here see the vera causa of one set of waterspouts.’

In Darwin’s response to Huxley’s critique of *Origin*, he also construes what he summarised as Huxley’s demand to prove natural selection a *vera causa* as a demand to produce what Huxley would recognise as ‘positive’ observational evidence (Sect. 3). Witness Darwin’s persistent efforts to produce such evidence from plant-experiments in his own garden, as well as from other horticulturalists and animal-breeders, to prove that domestic breeds had separated so far as to be ‘sometimes sterile with other breeds’ (e.g. Darwin, 1863b; see Sect. 3).

It is this observation-based sense of evidence which informs the chapter-plan of *Origin*. Because, of course, as the latter two-thirds of the postscript to Bentham confirm, *Origin*’s proposed ‘principle,’ ‘hypothesis,’ ‘theory’ or ‘general law’ of natural selection could never *be observed* to produce the detailed results Darwin’s book claimed that it *had* produced. The origination of new adaptations and new species was a process which the book held to take anything from ‘many thousands’ to ‘an almost infinite number’ of successive generations (Darwin, 1859a, pp. 114, 481).^10^ Hence, as when Lyell’s *Principles* set out the geological processes observable in the here and now by which he would explain the formation of geological features dating from the earth’s remotest past (Rudwick, 1970), *Origin* sets out from what *could* be empirically witnessed in order to deduce what *could not* be witnessed.

### *Origin*’s presentation of natural selection

*Origin*’s first three chapters elaborate several sets of empirical ‘laws,’ that is, statements based on repeated observations that describe (and thus predict) a ‘sequence’ of natural events: ‘laws of inheritance’ (Ch.1); laws of ‘variability’ and ‘correlated growth’ (Ch.2); and the law or ‘doctrine’ of Malthus, that populations of plants and animals have the reproductive capacity to increase at a ‘geometrical ratio,’ whilst food supplies, at best, increase at an arithmetical rate, resulting in a ‘struggle for existence’ (Ch.3). Each of these chapters details the many kinds of observable event which the said laws cover, giving copious examples. The laws themselves—whether of inheritance or variation—are, for ‘the most part unknown,’ or, at best, ‘dimly understood,’ *Origin* says (Darwin, 1876, pp. 9–10). Yet, the existence of the phenomena these laws are meant to describe is hard to question, given the detailed observations and experiments *Origin* recounts regarding: the facts of inheritance in domesticated varieties of animal and plant; the multitudinous variability or ‘individual differences,’ whether in wild species, subspecies and varieties or in domestic breeds; and of the various kinds of competition and mutual aid (as in ‘social plants’) entailed in what Darwin underlined was a ‘metaphorical’ struggle between members of the same and different species to survive, thrive, and reproduce (Darwin, 1859a, pp. 62–63, 70–71).

Whilst inheritance and variability were necessary preconditions for natural selection, its principle engine was the struggle for life, which would winnow the more useful variations in a given habitat from the less. This struggle was a theoretical construct in Malthus. The aim of *Origin*’s third chapter was to line up ‘better evidence on this subject than mere theoretical calculations, namely … numerous recorded cases,’ showing both species’ explosive potential for fecundity, and the vulnerability of individuals to a variety of environmental and inter-organism challenges (Darwin, 1859a, p. 64). Chapter Three particularly stresses the ‘web of complex relations’ between different creatures’ fates and those of the other organisms in their habitat (Darwin, 1859a, p. 73). Importantly, this metaphorical ‘struggle for life’ did not just betoken competition, but relative reproductive success and, the ‘dependence of one being on another,’ whether from different species (as with the symbiosis between moths and orchids), or from the same species, as with ‘social plants’ and ‘social animals’ who render ‘mutual aid’ to one another (Darwin, 1876, p. 50; Bradley, 2020).

The logical dependence of *Origin*’s fourth chapter, ‘Natural Selection’—and the book’s central thesis—on its first three chapters (on inheritance, variation, and the struggle for existence respectively) is reiterated throughout the book, from its first pages^11^:

As many more individuals of each species are born than can possibly survive; and as, consequently, there is a frequently recurring struggle for existence, it follows that any being, if it vary however slightly in any manner profitable to itself, under the complex and sometimes varying conditions of life, will have a better chance of surviving, *and thus* be naturally selected. From the strong principle of inheritance, any selected variety will tend to propagate its new and modified form (Darwin, 1859a, p. 5, my italics;).

Through exegesis of its central argument:

… it may be asked, how it is that varieties, which I have called incipient species, become ultimately converted into good and distinct species, which in most cases obviously differ from each other far more than do the varieties of the same species? How do those groups of species, which are called distinct genera, and which differ from each other more than do the species of the same genus, arise? *All these results … follow inevitably from the struggle for life* (Darwin, 1859a, p. 61, my italics)

To its last page:

It is interesting to contemplate a tangled bank, clothed with many plants of many kinds, with birds singing on the bushes, with various insects flitting about, and with worms crawling through the damp earth, and to reflect that these elaborately constructed forms, so different from each other, and dependent upon each other in so complex a manner, have all been produced by laws acting around us. These laws, taken in the largest sense, being Growth with reproduction; Inheritance which is almost implied by reproduction; Variability from the indirect and direct action of the conditions of life, and from use and disuse; a Ratio of Increase so high as to lead to a Struggle for Life, *and as a consequence to Natural Selection*, entailing Divergence of Character and the Extinction of less improved forms (Darwin, 1859a, pp. 489-490, my italics).

Such dependence is unavoidable, given the impossibility of gathering within a single human lifetime any eyewitness evidence for the efficacy of natural selection. This logic impels us to reject any claim that *Origin* makes ‘successive independent evidential cases … *for natural selection existing at present*’ (e.g. Hodge, 2000, p. 30). Such a statement would only make sense if natural selection *were already assumed to be the consequence* of the contributory laws copiously evidenced to ‘exist at present’ in the book’s opening three chapters—precisely the assumption writers like Hodge aim to overturn.

To underline this point, witness a crucial contrast between the first three chapters and the fourth. Unlike its predecessors, the supporting materials in Chapter Four ‘Natural Selection’ *are not* the fruit of first-hand observation, being presented entirely in the form of ‘*imaginary* illustrations’ (Darwin, 1859a, p. 90, my italics; Bradley, 2011).

It is not until the latter parts of the fourth chapter that *Origin* starts seriously to discuss the *causes* for the laws evidenced in the book’s first three chapters—*and thus* of natural selection. The causal mechanism producing the laws of *inheritance* (i.e. the transmission of heritable characters) is bypassed.^12^ Instead Chapters Four, Five (‘Laws of Variation’) and Six (‘Difficulties of the Theory’) proceed to explicate several causal processes that produce *variations*. Here, pride of place goes to the role of changed habits in directing selection. This theme stands out in Chapter Four’s illustrations of Darwin’s ‘principle of divergence’ of character (cf. adaptive radiation): *Origin* asks us to imagine the case of a carnivorous quadruped, ‘of which the number that can be supported in any country has long ago arrived at its full average’:

If its natural power of increase be allowed to act, it can succeed in increasing (the country not undergoing any change in conditions) only by its varying descendants seizing on places at present occupied by other animals: some of them, for instance, being enabled to feed on new kinds of prey, either dead or alive; some inhabiting new stations, climbing trees, frequenting water, and some perhaps becoming less carnivorous.

Darwin (1859a, p. 179; 1876, p. 8) formalized this habit-first causal process for the evolution of transformative adaptations—as when ‘a land carnivorous animal’ had been ‘converted into one with aquatic habits’—in a way that showed how ‘changed habits produce an inherited effect.’ *Origin*’s clearest example of this *non-Lamarckian* process highlighted how ‘transitional habits’ had plausibly resulted in the evolution of flying squirrels^13^:

*Origin* (Darwin, 1859a, pp. 179–186) asks us to imagine that, a long time ago, some adventurous, flightless squirrel-ancestors had formed a new habit of launching themselves, not just from branch to branch, but from tree-top to tree-top. Tree-surfing would put a new premium on glide-friendly changes to the squirrels’ physique (stronger spring at take-off, better depth vision, lighter body-weight, more aerodynamic tail, broader flanges of skin between front and back legs). Any chance heritable variation that fitted them better to their novel habit would have increased their reproductive success compared to unchanged conspecifics. Hence, ‘it would be easy for natural selection to fit the animal, by some modification of its structure, for its changed habits.’ Thus, while the production of what we now know as genetic variations—which must have stabilized the bodily changes that make tree-surfing easier for squirrels—might be random, the direction of adaptation would be set by the *non-random* agentic innovations of the ancestral squirrels.

‘Changed habits’ included ‘use and disuse,’ not just in animals but in plants. Of changed habits in plants, Darwin (1859a, pp. 139–143) cited the ‘acclimatisation’ of, for example, ‘the pines and rhododendrons, raised from seed collected by Dr. Hooker from trees growing at different heights on the Himalaya, [which] were found in this country to possess different constitutional powers of resisting cold’—seeds taken from higher in the mountains being found habitually more resistant to chilly British weather than their cousins from the mountains’ lower slopes. Darwin (e.g. 1859a, p. 76) typically framed the qualities of an organism in terms of ‘strength, habits, and constitution.’ The fate of variations in anatomy very often depend on an organism’s habits, according to his accounts, as, for example, with: the displays that feature sexual ornaments and the fights, which, he argued, must have led to the sexual selection of tusks and other weapons of sexual rivalry; the eating habits of birds with different shaped bills (e.g. finches in the Galapagos archipelago; Lindholm, 2015); insects’ adaptations to feeding from and so pollinating certain species of flower; closely-related animal species avoiding hybridisation by ‘haunting different stations’ of a given habitat; or the growth of hardness in pigeon chicks’ beaks (used for cracking their way out of their egg) (Darwin, 1859a, pp. 87, 103; 1882).

Other causes of variability proposed by *Origin* included ‘direct action of the environment’—on the ‘plastic’ (Darwin’s word: 1876, pp. 62, 106, 438) quality of the reproductive system, and the creature’s ‘whole organisation.’ Such action depended both on the nature of the organism and the nature of the conditions (e.g. climate, altitude), the nature of the organism being ‘much the more important,’ according to Darwin (1876, p. 6)—an emphasis now re-echoing through today’s post-MS biology with its so-called ‘return to the organism’ (e.g. Lewontin, 1983; Nicholson, 2014; Walsh, 2015).

Finally, the domain of phenotypic variability is not coextensive or neatly aligned with those ‘variations’ of relevance to a theory of natural selection. Not only may some of the ordinary doings of organisms fail to impinge on the struggle for existence—‘the war of nature is not incessant’ (Darwin, 1859a, p. 79). Even those that do so impinge may not result solely in ‘advantages,’ but also—as Chauncey Wright (1870, p. 293) argued—‘limiting disadvantages,’ likely to undermine fitness. *The Descent of Man* (Darwin, 1874, p. 571) proposed that Wright’s argument had ‘an important bearing on the acquisition by man of some of his mental characteristics’—citing in illustration how processes that (adaptively) ensured group cohesion, could simultaneously foster *maladaptive* customs and superstitions in some peoples. Examples included tribes where infanticide and cannibalism were customary, as reported by some ethnographers, plus, in Darwin’s own society, mating choices based on ‘mere wealth or rank’ (Darwin, 1874, pp. 121–122, 617).

## Discussion

Given Darwin’s identification of *verae causae* with processes that can be directly observed, it makes sense that he should have structured *Origin* to prove the existence of natural selection—something *unobservable*—as being a higher-order *consequence* of other *observable* (causal) processes. To recognise *Origin* presents natural selection as an effect of other causes, not a cause in its own right, is not merely a matter of textual exegesis, however. Such recognition has dramatic repercussions for the contemporary interpretation of evolutionary theory: because it fells the central pillar of gene-based MS constructions of the natural world—the belief that evolution is caused by natural selection. This forces on evolutionary scientists the need to seek a brand new conceptualisation of the relationship between evolution and what Walsh (2015, Ch.2.1) calls ‘the normal activities of organisms,’ including ourselves.

Here, the uptake of evolutionary theory by psychologists furnishes an apt illustration. Psychology is the central scientific site for examining the normal activities of organisms, particularly of human beings. So, how would adoption of the view that natural selection is ‘an analytic consequence’ (Walsh, 2015, Ch.2.1) of the normal lives of organisms alter contemporary evolutionary psychologies? Most significantly, it would disconnect how evolutionary science approaches the study of behaviour from any constraint by ideas about how natural selection operates (e.g. the need to calculate ‘inclusive fitness’; or to speculate about a prehistoric ‘environment of evolutionary adaptedness’; Buss, 2009; Tooby & Cosmides, 2016). Because, as Walsh (2015, Ch.2.1) says, ‘given the normal activities of organisms, nothing needs to be added [to our theoretical framework] to get populations to change in the ways that Darwin describes as natural selection.’ Which represents a complete reversal of those tenets of evolutionary psychology that produce statements like these (italics mine):

Like vision and language, our emotions and cognitive faculties are complex, useful, and non-randomly organized, which means that *they must be a product of* the only physical process capable of generating complex, useful, non-random organization, namely, *natural selection* (Pinker, 2005, p. xiv).

Because mental phenomena are the expression of complex functional organization in biological systems, *and complex organic functionality is the downstream consequence of natural selection*, then it must be the case that the sciences of the mind and brain are adaptationist sciences, and psychological mechanisms are computational adaptations (Tooby & Cosmides, 2016, p. 11).

[The brain’s] programs were designed not by an engineer, but by natural selection, *a causal process that retains and discards design features based on how well they solved adaptive problems in past environments* (Tooby & Cosmides, 2016, p. 19).

If natural selection is not an ‘upstream’ causal process which produces psychological phenomena, but a ‘downstream consequence’ of the normal activities of organisms—only *some of which* have adaptive consequences (cf. Chauncey Wright, above)—then our theoretical attention must switch from claims about natural selection, to the need adequately to conceptualise how agency manifests itself in the natural world. Perhaps we should not be surprised, therefore, that when we examine how Darwin himself presented his studies of various creatures’ ‘habits’ or behaviour—human group-processes and facial expressions; sexual displays in animals; worms’ intelligence and the motility of the ova of *Flustra*; mutual aid among social animals and the problem-solving movements of plant growth—we find these all reflect a single, coherent vision. According to Darwin (e.g. 1859a, p. 61), any studied habitat is maintained by the ‘infinitely complex’ web of actions and reactions linking the habits of the focal organism ‘to other organic beings and to external nature.’ It is this vision of the interdependencies created and maintained by agency which underpins how Darwin construed what he called ‘the struggle for life,’ and, as a consequence, how he understood natural selection (Bradley, 2020).

## Conclusion

The idea that natural selection is the causal force or mechanism which produces evolutionary adaptations and originates new species remains for many a scientific truism, thanks to the continuing appeal of ‘genes-eye’ MS accounts of evolution. This essay rejects a corresponding truism in Darwin scholarship, which holds that the main aim of *Origin*’s (Darwin, 1859a, p. 459) ‘one long argument’ is to prove natural selection the causal mechanism or *vera causa* responsible for the evolution of adaptations and new species.^14^ Specifically, I show how modern historiographic constructions of Darwin’s supposed authorial ‘intention,’ ‘desperation,’ or ‘commitment’ to prove natural selection a *vera causa* in *Origin* are built on an unnecessarily selective sample from what Darwin himself wrote about *verae causae*, typically highlighting just one remark, comprising the first third of Darwin’s (1863b) brief postscript to a letter to George Bentham.

My starting-point was different. I began by examining the context for *all* Darwin’s known uses of the term ‘*vera causa*.’ From this beginning I have argued that, provided one grounds one’s views of *Origin*’s arguments upon: how Darwin *himself* used the term *vera causa* (as requiring first-hand observational evidence); how this usage conforms to the commonest meaning of the term among Victorian philosophers of science; how *Origin* itself sets up, and repeatedly restates, the logical dependence of natural selection on inheritance, variation and the struggle for life; how, responding to criticism early in 1860, Darwin disputed the need to prove natural selection the true cause of adaptive change and evolution; and how that dispute led him to revise later editions of the book—then one must conclude that, according to Darwin, natural selection is an effect of other causes, not a cause in its own right.

One advantage of recognising that *Origin* does not comprise just one argument—aimed at proving natural selection the true cause of adaptation—is to re-focus historiographic and scientific attention on all the other arguments that the book makes. Several of these arguments have become central to evolutionary science over the last twenty years, though often without any awareness by modern scientists of antecedent arguments in *Origin*.^15^ These include the leading role played by organisms’ agency in the genesis of adaptations (cf. ‘transitional habits’ aka the ‘Baldwin effect,’ ‘genetic accommodation,’ and ‘niche construction’: Darwin, 1876, pp. 138–143; Gould, 2002, pp. 125–127; Noble & Noble, 2017; Noble, 2021; Odling-Smee et al., 2003; Walsh, 2015); the importance of plasticity of structure in the evolution of new adaptations (Darwin, 1876, pp. 62, 106, 438; West-Eberhard, 2003; 2008); the direct effect of external conditions (Darwin, 1876, p. 67; Gilbert & Epel, 2015, pp. 435ff); the recognition that ‘inheritance’ includes ‘two distinct processes’—the transmission of heritable characters from parent to offspring *and their development* (Darwin, 1876, pp. 114–15, 119–122; 1874, p. 227; Walsh, 2010); and multi-level selection (Darwin, 1876, pp. 67–68; Wilson & Wilson, 2007).

As soon as contemporary scientists accept that, as per Darwin’s argument in *Origin*, natural selection does not cause, but *results from* the ordinary activities of organisms, contemporary evolutionary theorists must address a new foundational challenge: the need to construct a viable, evidence-based picture of the natural world as what I have called a ‘theatre of agency’ (Bradley, 2020). Only when they have such a picture will scientists be in a position to work up an intelligible account of natural selection. The pioneering instance of such a working-up constitutes a theme central to Darwin’s many publications.

## Footnotes

1 E.g. the third edition of *Origin* (Darwin, 1861a, p. 88, my italics) adjusted the above quotation to begin: ‘*It may metaphorically be said that* natural selection is daily and hourly scrutinising, throughout the world …’.

2 Physicists still debate what causes the phenomena the law of gravity describes. At least fourteen theories of gravity are currently in play.

3 NB Darwin here denies that the undulatory theory of light identifies a ‘vera causa’ on the grounds that ethereal undulations had never been observed. In this he equates *verae causae* with demonstration by positive observational evidence (as discussed later in this section). Conversely, Darwin also implies that causal hypotheses (e.g. ‘interference patterns result from the wave-form of light’) do not have to equate to *verae causae*. Hence the need for my examination of the way the argument in *Origin* is presented to prove my case that he cast natural selection as a consequence of other causes (see Sect. 5.2 below).

4 Note that controversy over the causal status of natural selection was still live in 1870: ‘Strictly speaking, Natural Selection is not a cause at all, but is the mode of operation of a certain quite limited class of causes’: Chauncey Wright, ‘Review [of Contributions to the Theory of Natural Selection. A Series of Essays by Alfred Russell Wallace],’ North American Review111, (1870), p. 293.

5 It is notable that this postscript is *never* quoted in full by the authors I have cited. All stop before the words, ‘When we descend to details …’ Even I have not quoted its two final sentences, viz. ‘The latter case seems to me hardly more difficult to understand precisely & in detail than the former case of supposed change. Bronn may ask in vain the old creationist school & the new school why one mouse has longer ears than another mouse—& one plant more pointed leaves than another plant.'

6 Hull (1973, pp. 7–8; 2003, pp. 185–188) argues that Darwin was mistaken in this belief, at least where Mill was concerned.

7 Albeit in its second paragraph, which historiographers seldom if ever quote or discuss—as noted previously.

8 A contributing factor here may have been Darwin’s growing sense that his Glen Roy (1839) paper had been a ‘gigantic blunder.’ According to Barrett (1973, p. 24) Darwin came to the conclusion that it was ‘metaphysics’ that ‘had tripped him.’ Hence his determination that this ‘didn’t happen again.’.

9 This eruption gave birth to Sabrina Island.

10 This difficulty was even more acute than with Newton’s law of gravity. Yes, gravity was indeed invisible, and had an unknown causal mechanism. But its putative effects can be observed and measured here and now: pendulums swinging; apples accelerating towards earth; tides rising and falling; planets orbiting. Natural selection could neither be observed in itself. Nor could the immensely slow production of its putative effects be observed in the span of any one human lifetime.

11 Or, indeed, arguably *from its title*, which equates, ‘the origin of species by means of natural selection,’ with ‘the preservation of favoured races in the struggle for life.’

12 A causal mechanism for inheritance is tentatively presented in the last chapter of *Variation under Domestication* (Darwin, 1868), titled: ‘Provisional hypothesis of pangenesis,’ though this hypothesis was never incorporated or mentioned in later editions of *Origin.*

13 This process was supposedly only discovered after Darwin’s death, by James Mark Baldwin in 1896 (similar formulations were advanced at about the same time by Conway Lloyd Morgan and others). As a result it is now known as ‘the Baldwin effect’ (after Simpson, 1953). However, Baldwin (1909, p. 11) explicitly denied that he had discovered this process, which *he* called ‘organic selection,’ because, he said, *Origin* had clearly described it in 1859, under the heading ‘transitional habits.’

14 The very idea that this claim of Darwin’s (1859a, p. 459)—that *Origin* comprised ‘one long argument’—espoused a sober epistemological commitment, arguably misrecognises its rhetorical, not to say wishful, status. In her commentary, Beer’s (2000, pp. 42–43) reminds us how *Origin*’s ‘argument’ does not proceed according to an epistemological schema, but rather by laboriously accumulating ‘an unruly superfluity of material’ through ‘a strange intermingling of acquisition, concretion, analogy and prophecy.’ Hence, it is ‘only gradually and retrospectively,’ she says, that ‘the force of the [‘one long’] argument emerge[s] from the profusion of example.’.

15 There are several exceptions to this claim, most notably for me; Mary Jane West-Eberhard (2003, 2008).
